# Long-term outcome of a child with hyperinsulinism- hyperammonaemia syndrome

**DOI:** 10.1186/1687-9856-2013-S1-P182

**Published:** 2013-10-03

**Authors:** HC Yau, GWK Wong

**Affiliations:** 1 Department of Paediatrics, Prince of Wales Hospital, The Chinese University of Hong Kong, Hong Kong SAR, China

## Aims

Clinical features of hyperinsulinism-hyperammonaemia syndrome are variable. A potential genotype-phenotype correlation of GLUD1 mutation and clinical features has been suggested. We here report the long-term outcome of a child with hyperinsulinism-hyperammonaemia syndrome.

## Methods

A male infant was born at 38 gestational weeks with birth weight of 3.605 kg. He presented with convulsion at 28 days of life. Physical examination was unremarkable. He was initially treated as meningoencephalitis. CT brain was unremarkable. Bacterial and viral cultures were negative. Plasma glucose upon admission was 1.2mmol/l with negative urine ketone. Glucose infusion was high up to 12.1 mg/kg/min. Two sets of critical samples were obtained during hypoglycaemia.

**Table 1:** 

Date	28 days of life	34 days of life
**Plasma glucose (mmol/l)**	1.9	2.0

**Insulin (mIU/l)**	23.9	10.5

**Growth hormone (IU/l)**	54	

**Cortisol (nmol/l)**	311	

Workup for metabolic diseases was unremarkable. But ammonia levels were persistently high up to 252-353 umol/l. Octreotide injection was started to achieve euglycaemia. Protein free diet was tried but without success in lowering ammonia levels. Octreotide was later switched to oral diazoxide and hydrochlorothiazide. Full enteral feeding was established with stable glucose levels.

## Results

Genetic analysis showed a mutation in axon 10;Asn410I1e (N410I), a mutation of AAC to ATC at codon 410. The diagnosis of hyperinsulinism-hyperammonaemia syndrome was confirmed. We follow the child until he is 6 years of age. Home blood glucose monitoring prevented him from hypoglycaemia. Fasting test was performed every year to fine tune his diazoxide dosage. He remained seizure free while ammonia levels remained high up to 123-218 µmol/l. Formal developmental assessment performed at 5 years of age showed delay in cognitive and speech development for 1-1.5 years, and attention-deficit hyperactivity syndrome.

## Conclusion

Clinical features of hyperinsulinism-hyperammonaemia syndrome are variable. A genotype-phenotype correlation requires confirmation in larger series of patients.

